# P-1885. Online Medical Education Effectively Improves Clinician Knowledge, Competence and Confidence with Implementing Antimicrobial Stewardship When Testing and Treating Suspected Upper Respiratory Infections with Available Molecular Rapid Diagnostic Tests

**DOI:** 10.1093/ofid/ofaf695.2054

**Published:** 2026-01-11

**Authors:** Arun S Nair, Roderick Smith, James Martorano

**Affiliations:** Medscape Education, TARRYTOWN, New York; Medscape, New York, New York; Medscape EDucation, New York, NY

## Abstract

**Background:**

This study evaluated the impact of online CME on clinician knowledge, competence, and confidence in using molecular rapid diagnostic tests (mRDTs) and antimicrobial stewardship (AMS) for diagnosing and managing suspected upper respiratory infections (URIs).

**Methods:**

The educational activity included 5 video-based micro-CME chapters presented by 3 experts, allowing learners to select chapters. Educational impact was assessed using a repeated paired pre-/post-assessment design, with participants serving as their own controls. McNemar’s tests (*P* < .05) determined statistical significance in mastery (correct decisions or improved confidence). A confidence-based assessment (CBA) evaluated changes in competence and confidence, categorizing responses as mastery (correct & confident), doubt (correct but not confident), uninformed (incorrect & not confident), or misinformed (incorrect but confident). One question per chapter was included. The activity launched 7/2023, with data collected until 9/2024.

**Results:**

By 4/2025, the curriculum reached ∼4,300 learners, primarily Infectious Disease (ID) Specialists, General Practitioners (GPs), and Emergency Medicine (EM) Physicians. Pre-assessment knowledge, competence, and confidence in implementing mRDTs and AMS practices averaged ∼55% across specialties. Post-education, significant improvements of 54%, 55%, and 56% were observed among ID, GP, and EM learners, respectively (*P* < .001).

Additional learner specific insights from the assessment include:

• In a case scenario, ∼50% of learners started empiric antibiotics despite the patient presenting well and no positive test results, highlighting persistent antibiotic misuse learning gaps

• Despite a 47% average improvement in AMS across target groups, ∼50% of learners still lack mastery in applying stewardship principles when managing suspected upper respiratory infections with available mRDTs

**Conclusion:**

These findings demonstrate that online CME improves understanding of mRDTs and AMS strategies for diagnosing and managing suspected URIs. Future education should include case scenarios and emphasize real-world practices, focusing on when to use mRDTs and how to integrate AMS principles for suspected infections in vulnerable patients populations.

**Disclosures:**

All Authors: No reported disclosures

